# Water Intake in Pregnant Women in China, 2018: The Report of a Survey

**DOI:** 10.3390/nu13072219

**Published:** 2021-06-28

**Authors:** Ye Ding, Zhencheng Xie, Xiaolong Lu, Hongliang Luo, Han Pan, Xiaofang Lin, Jieshu Wu, Zhixu Wang

**Affiliations:** 1 Department of Maternal, Child and Adolescent Health, School of Public Health, Nanjing Medical University, Nanjing 211166, China; dingye@njmu.edu.cn (Y.D.); zhenchengxie@njmu.edu.cn (Z.X.); lu123xiaolong@163.com (X.L.); seattle_lxf@163.com (X.L.); jwu@njmu.edu.cn (J.W.); 2 Danone Open Science Research Center for Life-Transforming Nutrition, Shanghai 201204, China; Hongliang.LUO@danone.com (H.L.); Zoe.PAN1@danone.com (H.P.)

**Keywords:** total water intake, water from beverages, water from foods, adequate intake, pregnant women

## Abstract

Background: Adequate water intake in pregnant women plays an important role in their health and in fetal growth and development. However, there is insufficient applicable data to guide and evaluate the water intake of pregnant women in China. Based on a nationwide sample of pregnant women, we mainly aimed to investigate the daily total water intake (TWI) and the contribution of different beverages and food sources to the TWI, to assess the percentage of participants who comply with the adequate intake (AI) value of water set by the Chinese Nutrition Society (CNS) and the European Food Safety Authority (EFSA) and to analyze the contribution of different water sources to the daily total energy intake (TEI). Methods: A multi-stage sampling method was used to recruit pregnant women from 11 provinces and two municipalities in China. A 4-day online diary with a food atlas was used to assess water and dietary intake. Finally, 653 pregnant women were included in the analysis. The Mann–Whitney U test and the independent-sample t-test were used to compare the differences between related variables in different age groups or different gestational periods, and partial correlation was used to explore the correlation between water and energy intake. Results: The median daily TWI of pregnant women was 2190 mL, of which water from beverages and foods accounted for 52.9% and 47.1%, respectively. Approximately 80.5% of the water from beverages was mainly from plain water (*r* = 0.973), while in the part of the water from foods, dishes (32.4%) were the main contributors (*r* = 0.663). Only 16.4% and 43.8% of the total population met the TWI recommendation set by the CNS and EFSA, respectively. Among these, the contribution of the water from beverages was higher than that of the water from food. For those whose TWI did not reach the recommended level, the contribution of the water from beverages was almost equal to that of food. The median daily TEI of pregnant women was 1589 kcal, of which beverages accounted for 9.7%. Milk and milk derivatives (71.3%) were the main contributors to energy from beverages, accounting for 71.3% (*r* = 0.444). Although sugar-sweetened drinks only accounted for 10.1% of the energy from beverages, they were highly correlated with energy from beverages (*r* = 0.836). Through grouping analysis, age and gestational period had no significant effect on the above main results. Conclusions: This was the beginning of a nationwide study on the TWI of pregnant women in China, and the results provide evidence of the need for interventions to improve water intake among pregnant women and the revision of reference values for AI of TWI in pregnant women in China.

## 1. Introduction

Water is the main component of human tissue and is involved in a series of physiological functions, such as maintaining normal osmotic pressure, electrolyte balance and body temperature [1]. Insufficient water intake or excessive water loss can cause dehydration, which has adverse effects on body physiology and cognitive function [2,3]. During pregnancy, special physiological changes, for example, increased maternal total body water, blood volume, cardiac output, and blood flow to the kidneys and uteroplacental unit [4,5,6], cause increased water requirements, accompanied by increased water loss, ultimately putting pregnant women at a high risk of dehydration [7]. There is evidence that dehydration in pregnant women could have a negative impact on maternal and infant health, which could lead to constipation, oligohydramnios, preeclampsia and some other pregnancy complications, and even increase the risk of adverse pregnancy outcomes such as abortion and preterm delivery [8,9,10,11]. Therefore, pregnant women need adequate water intake to maintain normal hydration.

As mentioned in the previous review [12], to promote appropriate water intake in pregnant women, many countries have set adequate intake (AI) for the total water intake (TWI) of pregnant women. In general, compared with non-pregnant women, the AI of TWI for pregnant women is increased. For example, the European Food Safety Authority (EFSA) recommended 2.3 L/day for pregnant women [13]. The development of reference values for the AI of TWI is based, at least in part, on adequate survey data. However, there was insufficient data to deduce the TWI of pregnant women in China. To the best of our knowledge, only one study has reported the TWI for pregnant women in Beijing [14], China, which is not representative. Given the fact that energy intake increases by 300 kcal/day during gestation, it is suggested that pregnant women should increase their water intake by 300 mL/day relative to non-pregnant women. In 2013, according to the reference value of the United States, the Chinese Nutrition Society (CNS) added 300 mL/day to the recommendation for non-pregnant women and recommended that the AI of TWI for pregnant women be set at 3.0 L/day [15]. However, there is insufficient data to determine whether the recommendation is suitable for pregnant women in China. Therefore, it is necessary to conduct a nationwide survey on the TWI of pregnant women in China.

The TWI is the sum of water from beverages and foods. The availability of beverages and foods, cultural factors, age and different physiological periods will influence the selection of beverages and foods, and the water intake obtained from beverages and foods can vary between countries and individuals. Some surveys on the water intake for adults in China showed that the water from foods accounted for 40–50% of the TWI, whereas it only accounted for 20–30% in European and American countries [16,17,18]. Compared with non-pregnant women, pregnant women need to eat more food to obtain sufficient energy and nutrients to promote maternal health and fetal growth and development, which will affect the water from foods and change the contribution of different sources of water to the TWI. Currently, there are few surveys on the water intake of pregnant women, which were carried out only in Greece, Mexico, Indonesia and China [14,19,20,21], and most of them only focused on liquid intake without information on water intake from foods. Although the TWI from beverages and foods was considered in the survey in Beijing [14], China, as mentioned above, the results of this study only reflect the water intake of pregnant women in the developed cities of northern China. Further surveys on this issue in other regions of China are needed to obtain more reliable and representative data.

Therefore, the primary aim of this study was to report the usual daily TWI and total energy intake (TEI: the sum of energy from beverages and foods) and the contribution of different beverages and food sources to the TWI and TEI of pregnant women according to age and gestational period in a nationwide sample in China in 2018. The secondary aim was to compare the TWI with the AI value set by the CNS and EFSA according to age and gestational period, and to investigate the contribution of the water from different beverages and food sources to the TWI according to whether the TWI reached the recommended threshold value. The final aim was to analyze the correlation between the water and energy intake of different sources and TWI, total fluid intake (the sum of the volume of different types of beverages), water from beverages and foods, TEI and energy from beverages and foods.

## 2. Materials and Methods

### 2.1. Study Participants

Our study was based on a cross-sectional survey of pregnant women in China (2018–2019). The participants were selected using multistage random sampling. There are 23 provinces, five autonomous regions, four municipalities and two special administrative regions in China. Based on geographical location, economic status and annual live birth rate, 11 provinces and two municipalities, including Guangdong, Sichuan, Jiangsu, Fujian, Yunnan, Hebei, Hubei, Anhui, Liaoning, Zhejiang, Henan, Shanghai and Beijing, were selected. Each region was then divided into urban and rural areas, and a survey city was selected for each area. Finally, participants were randomly recruited from each city based on the information provided by the Maternal and Child Health Centre (China) where they received public antenatal care. Prior to face-to-face interviews, the interviewers communicated with the participants by phone to determine the location of the data collection (usually in the participant’s home). Before the interview, the participants had a detailed understanding of the purpose and methods of the study and signed an informed consent form. This study was approved by the ethics committee of the Nanjing Medical University (Nanjing, China), ethic approval number is 2018-1123.

Healthy women with singleton pregnancies, aged 20–44 years, with no clinical diagnosis of infectious disease, no metabolic disorders (obesity, hypertension, diabetes, etc.), no pregnancy complications (hyperemesis gravidarum, gestational hypertension, gestational diabetes, etc.) and no malnutrition disorders (osteoporosis, anemia, iodine deficiency goiter, etc.) were eligible for inclusion in the present study. Pregnant women who could not report their water and dietary intake because of limited cognitive capacity and pregnant women who underwent in vitro fertilization, chemotherapy, or were involved in other studies on water or food restriction, were excluded. In general, women would not know that they are pregnant until 6–7 weeks of pregnancy because of the absence of their menstruation. Then, they will experience early pregnancy reactions with nausea, vomiting, loss of appetite and other symptoms, which will last until approximately 12 weeks of pregnancy. Both of these factors lead to difficulties in recruiting pregnant women in the first trimester (within 12 weeks) and affect the accuracy of water and dietary intake. Therefore, our study only included pregnant women in the second trimester (13–27 weeks) and the third trimester (28 weeks to pre-delivery).

The sample size of our study was determined using the formula: $N=\frac{{\left(Z_{\alpha }\times S\right)}^{2}}{d^{2}}$. *Z* is the value associated with the desired confidence level, and the confidence level was set at 95% ($Z_{\alpha }$ = 1.96) in our study. *S* is the estimated standard deviation of the population. In our study, this value was the result of a previous study, which showed that the standard deviation of daily plain water intake for pregnant women was 711 mL [14]. *d* is the maximum measurement error allowed by us, which is 100 mL. It was calculated that at least 195 participants were required for this study.

### 2.2. Water and Dietary Data Collection and Analysis

An online diary with Tablet/iPad/Phone resources was used to assess the water and dietary intake of pregnant women for four days (including two workdays and two weekend days). The food atlas of 303 types of food and two types of fluid containers were provided as visual aids to help participants estimate their water and dietary intake accurately [22]. The food list in the online diary included approximately 328 different types of food and beverages. More details were provided in our previous reports [23]. The data were collected by third parties (Danone Open Science Research Centre (Shanghai, China) and Taylor Nelson Sofres (Shanghai, China)). During the interview, well-trained interviewers asked participants to report all foods and beverages. 

The beverages collected were classified into six categories: plain water (tap and bottled water); hot beverages (coffee and tea); milk and milk derivatives (MMDs) (liquid milk, powdered milk, yoghurt, etc.); fruit and vegetable drinks (FVDs) (bottled or freshly squeezed), sugar-sweetened drinks (SSDs) (carbonated beverages, flavored beverages, functional beverages, milky tea, etc.) and botanical protein drinks (BPDs) (soybean milk, almond juice, walnut juice, etc.). The water from foods was classified into five categories: staple food, dishes (vegetables, beans and legume products, aquatic products, livestock meat, poultry and eggs), porridge, soup and snacks. A more detailed classification of beverages and water from foods is found in Supplementary Materials Tables S1 and S2.

Three variables were used to assess the water intake in our study: (1) water from beverages; (2) water from foods; (3) TWI, that is, the sum of the water from beverages and foods. The proportion of water content and energy for each food was established from the Chinese Food Composition Table [24]. An Excel worksheet was used for water and energy conversion and calculations. The main reference levels were based on AI values set by the CNS and EFSA by different ages and pregnancy periods.

### 2.3. Quality Control

A pre-survey was conducted to test the feasibility of the online diary. All investigators must be trained on the technical aspects of the survey before the study. Investigators were on hand to help participants use the online diary to maintain a high participation rate and avoid copying the previous day’s data into the next day’s record. The responses were retrieved and reviewed in time to ensure the completeness and validity of the results, and the errors were corrected.

### 2.4. Statistical Analysis

Data were entered into an Excel sheet from the four-day diaries related to food items in the food list. Statistical analysis of all data was performed using the SPSS software package version 26.0 (IBM, New York, NY, USA). The participants were grouped according to the upper limit of the best childbearing age (28 years) and different gestational periods (the second and third trimesters). Normally distributed continuous variables were expressed as the mean and standard deviation (SD), and non-normally distributed continuous variable data are expressed as the median and interquartile range. Categorical variables are expressed as frequencies (*n*) and percentages (%). The Mann–Whitney U test and independent-sample t-test were used to compare the differences between related variables in different age groups or different gestational periods. After adjusting for potential covariates, partial correlation was used to explore the correlation between total water intake, total energy intake and beverage and food intake in various categories. Values of *p* < 0.05 were considered statistically significant.

## 3. Results

### 3.1. Characteristics of the Study Participants

First, 680 pregnant women from 11 provinces and two municipalities were investigated for the study. Then, 27 participants who had reported too little or too much mean total water intake (less than 600 mL/day or more than 5000 mL/day) or mean total energy intake (less than 800 kcal/day or more than 3500 kcal/day) were excluded. Finally, a total of 653 pregnant women (mean age: 28.51 ± 3.86 years; range: 20–44 years) were included in the analysis. Among them, 307 (53.0%) and 346 (47.0%) pregnant women were in the second and third trimesters, respectively, which met our expectations (see the sample size in the Methods section for details).

### 3.2. Daily TWI from Different Sources

The median daily TWI of the total population was 2190 mL, of which 1145 mL was from beverages and 1011 mL was from foods, accounting for 52.9% and 47.1% of the TWI, respectively. Further analysis of the water from beverages showed that plain water (80.5%) was the main contributor, with a median daily intake of 1000 mL, followed by MMDs, BPDs, SSDs and FVDs, accounting for 14.7%, 2.6%, 1.4% and 0.8% of the water from beverages, respectively. In the portion of the water from foods, dishes (32.4%) were the main contributors, with a median daily intake of 313 mL, followed by staple food, snacks, porridge and soup, which accounted for 23.5%, 14.9%, 14.7% and 14.6% of the water from foods, respectively. 

As shown in Table 1, daily TWI and the contribution of water intake from different sources among Chinese pregnant women in 2018 were further compared by age and gestational trimesters. After grouping by age, there was no statistical difference in TWI, total fluid intake and water intake from different beverages between the two age groups. With regard to water from foods, the difference between the two age groups was also not statistically significant, except for the water from dishes, which showed that the absolute value and proportion of daily water intake from dishes in the high-age group were significantly higher than those in the low-age group (*p* < 0.05). Similarly, after grouping by gestational trimesters, there were no statistical differences in TWI, total fluid intake and water intake of different beverages and foods between the two groups.

### 3.3. Comparisons with the Adequate Total Water Intakes Set by the CNS and EFSA

**Figure 1:**
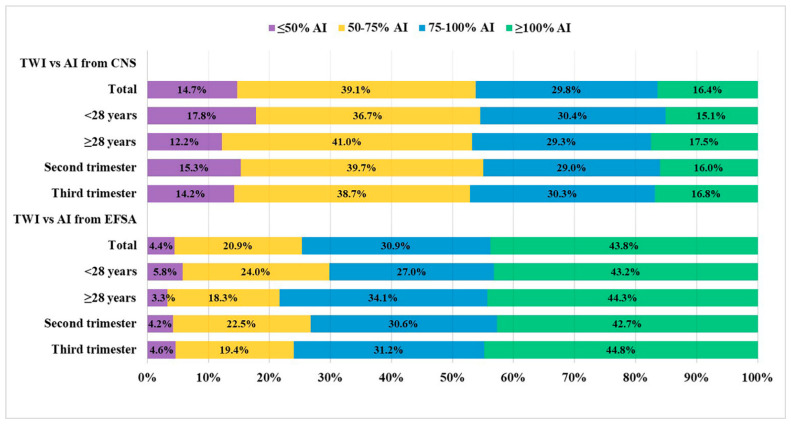
Percentages (%) of Chinese pregnant women in 2018 according to adherence categories of adequate intakes of total water intake set by the Chinese Nutrition Society or European Food Safety Authority categorized by age and gestational trimesters. AI: adequate intake; TWI: total water intake; CNS: Chinese Nutrition Society; EFSA: European Food Safety Authority.

Figure 1 shows the proportion of participants by age and pregnancy period, consuming ≥100%, 75–100%, 50–75% and ≤50% of the AI of TWI set by the CNS or EFSA. Compared with the AI of TWI for pregnant women set by the CNS, only 16.4% of the total population met the TWI recommendation. After grouping by age and pregnancy period, the results showed that the proportion of participants whose TWI reached the recommended threshold value set by the CNS in the high-age group was higher than that in the low-age group (17.5% vs. 15.1%), and this proportion in the third trimester was slightly higher than that in the second trimester (16.8% vs. 16.0%); however, these differences were not statistically significant. Compared with the AI of the TWI for pregnant women set by the EFSA, 43.8% of the total population met the TWI recommendation, which was significantly higher than the result compared with the recommendation set by the CNS (*p* < 0.05). After grouping by age and pregnancy period, the results compared by the recommendation set by the EFSA showed that the proportion of participants whose TWI reached the recommended threshold value in the high-age group was slightly higher than that in the low-age group (44.3% vs. 43.2%), and this proportion in the third trimester was higher than that in the second trimester (44.8% vs. 42.7%); however, these differences were not statistically significant.

As shown in Table 2, the total population was divided into different groups according to whether their TWI reached the AI threshold value set by the CNS or the EFSA, and the daily TWI and contribution of water intake from different sources were compared. When comparing actual TWI with the AI value set by the CNS, the median daily TWI of the group whose daily TWI reached the recommended value (*n* = 107, named group C1) was 3425 mL, and the contribution of the water from beverages was higher than that of the water from foods, which were 2071 mL (60.7%) and 1330 mL (39.3%), respectively. The median daily TWI (2057 mL) of the group whose daily TWI did not reach the recommended value (*n* = 546, named group C2) was much lower than that of group C1, and the source of TWI in this group was different from that of group C1. The contribution of the water from beverages was almost equal to that of the water from foods, which were 1044 mL (51.4%) and 952 ml (48.6%), respectively. Further analysis of the water from beverages showed that the main differences between the two groups were that the water from plain water and MMDs in group C1 was more than that in group C2 (1800 mL vs. 800 mL and 181 mL vs. 138 mL, respectively), and the contributions of SSDs and BPDs to water from beverages in group C1 were lower than those in group C2 (0.8% vs. 1.5%, 1.6% vs. 2.8%, respectively). These differences were statistically significant (*p* <0.05). While in the part of the water from foods, the water of staple food, dishes, porridge, soup and snacks in group C1 were all significantly higher than those in group C2 (*p* < 0.05).

When comparing actual TWI with the AI value set by the EFSA, the median daily TWI of the group whose daily TWI reached the recommended value (*n* = 286, named group E1) was 2805 mL, and the contribution of the water from beverages was higher than that of the water from foods, which were 1673 mL (57.2%) and 1224 mL (42.8%), respectively. The median daily TWI (1817 mL) of the group whose daily TWI did not reach the recommended value (*n* = 367, named group E2) was much lower than that of group E1, and the source of TWI in this group was different from that of group E1. The contribution of the water from beverages was almost equal to that of the water from foods, which were 854 mL (49.5%) and 857 mL (50.5%), respectively. Further analysis of the water from beverages showed that the main differences between the two groups were that the water from plain water and MMDs in group E1 was higher than that in group E2 (1500 mL vs. 700 mL and 191 mL vs. 117 mL, respectively), and the contribution of BPDs in water from beverages in group E1 was lower than that in group E2 (2.0% vs. 3.2%). These differences were statistically significant (*p* < 0.05). While in the part of the water from foods, the water of staple food, dishes, porridge, soup and snacks in group E1 were all significantly higher than those in group E2 (*p* < 0.05).

### 3.4. Daily TEI from Different Sources

The median daily TEI of the total population was 1589 kcal, of which 128 kcal was from beverages, and 1420 kcal was from food, accounting for 9.7% and 90.3% of the TEI, respectively. Further analysis of the energy from beverages showed that MMDs (71.3%) were the main contributors, with a median daily intake of 96 kcal, followed by BPDs, SSDs and FVDs, accounting for 10.2%, 10.1% and 5.2% of the energy from beverages, respectively. In terms of the energy from foods, dishes (50.3%) were the main contributors, with a median daily intake of 713 kcal, followed by staple food (447 kcal), snacks (205 kcal) and porridge (14 kcal), accounting for 31.5%, 15.6% and 2.7%, respectively, of the energy from foods.

As shown in Table 3, the daily TEI of different sources among Chinese pregnant women in 2018 was further compared by age and gestational trimesters. After grouping by age, TEI, energy from foods and energy from dishes in the high age group were significantly higher than those in the low age group, and the differences were statistically significant (*p* < 0.05). After further statistical analysis according to different categories of beverages and foods, there were no significant differences in energy intake of different beverages and foods (except dishes) between the two age groups. Regarding the contribution of the energy intake from different sources, the differences in TEI and energy intake of different beverages and foods between the two age groups were not statistically significant. After grouping by gestational trimesters, there were no statistical differences in TEI and energy intake of different beverages and foods between the two groups except for the proportion of the energy from FVDs, which showed that this proportion in the third trimester (6.2%) was higher than that in the second trimester (4.0%), and the difference was statistically significant (*p* < 0.05).

### 3.5. Partial Correlation Analysis

In Table 4, the correlations between the water and energy intake of different sources and TWI, total fluid intake, water from beverages and foods, TEI and energy from beverages and foods (adjusted for age and gestational period) were assessed. As expected, the TWI was highly correlated with total fluid intake, water from beverages and plain water (*r* = 0.874, 0.871 and 0.829, respectively), and was moderately correlated with water intake from foods (*r* = 0.609). The TWI is the sum of the water from beverages and foods. Further analysis found that the water from beverages was highly correlated with plain water (*r* = 0.973), and the water from foods was moderately correlated with water from staple food and dishes (*r* = 0.526 and 0.663, respectively). With regard to energy intake, The TEI was highly correlated with energy from total foods (*r* = 0.946) and moderately correlated with energy from stable food, dishes and snacks (*r* = 0.559, 0.722 and 0.628, respectively). Similarly, the TEI is the sum of the energy from beverages and foods. Further analysis found that energy from beverages was highly correlated with energy from SSD (*r* = 0.836), and energy from foods was moderately correlated with energy from stable food, dishes and snacks (*r* = 0.638, 0.744 and 0.633, respectively). In the correlation analysis of the energy intake and water intake, it was found that the TEI was moderately correlated with water from total foods and dishes (*r* = 0.616 and 0.560, respectively), energy from beverages was moderately correlated with water from SSD (*r* = 0.725), and energy from foods was moderately correlated with water from total foods and dishes (*r* = 0.647 and 0.585, respectively).

## 4. Discussion

To our knowledge, based on a nationwide sample in China, this is the first survey to investigate the daily TWI and TEI and the contribution of different sources of beverages and foods to the TWI and the TEI in pregnant women and to assess the percentage of participants who comply with meeting the recommended AI value of water set by the CNS and the EFSA.

The findings of this study showed that the median daily TWI of pregnant women in China was 2190 mL (including 1145 mL of water from beverages and 1011 mL of water from foods), which is lower than 2539 mL (including 1000 mL of plain water, 178 mL of water from other beverages and 1121 mL of water from foods) previously reported in Beijing [14], China. This difference might be related to the representativeness of the participants. A previous study carried out in Beijing only represented the TWI of pregnant women in developed cities in northern China. Our study strictly followed the national multistage random sampling in China, reflecting the TWI of pregnant women in China, taking into account different geographical locations and socioeconomic levels. To the best of our knowledge, only three other countries have conducted surveys on the amount of water intake for pregnant women, of which only Greece [19] investigated the TWI, while those of Mexico [20] and Indonesia [21] only focused on the TFI. Specifically, the median daily TWI of pregnant women in Greece [19] was 2917 mL (including 1140 mL of plain water, 678 mL water from other beverages and 680 mL of water from foods); these figures were higher than the ones found in our results. The mean daily TFI of pregnant women in Mexico and Indonesia were 2620 mL and 2229 mL, respectively, which were also higher than the 1165 mL reported in our study. Only hypotheses could be made to explain these inter-country differences. The survey design, sampling method, race, age, season, geographical location, climate, culture factors, eating habits and the methods used to cook food and assess dietary intake could be possible explanations.

It is important to note that, in our study, the contribution of the water from beverages and foods to the TWI was relatively close, at 52.9% and 47.1%, respectively, which is similar to the findings of a previous study carried out on pregnant women in Beijing [14], China (52% and 48%, respectively) and female adults in Italy [25] (56% and 44%, respectively). However, the contribution of the water from beverages and foods to the TWI of female adults in other countries showed that the amount of water from beverages is much greater than that from foods, such as France [26] (62.2% and 37.8%, respectively), Balearic Islands [27] (65.4% and 34.6%, respectively), Spain [28] (67.9% and 32.1%, respectively) and Australia [29] (75.4% and 24.6%, respectively). This could be explained by differences in cultural factors, eating habits and cooking methods. Tubers, vegetables and fruits are the main sources of traditional Chinese food, which have high water content [30]. Meanwhile, as our results show, dishes and staple food were the main contributors of the water from foods of Chinese pregnant women, and their water content was moderately correlated with that in foods (r = 0.663 and 0.526, respectively). The cooking methods for these kinds of foods in China are mainly steaming and stewing, which largely conserves the water in such food. However, in Western countries, animal food and flour are the main food sources, of which water content is relatively low, and they are mainly cooked by baking, roasting and frying, which leads to the loss of water in such food [30,31]. In addition, Chinese residents have a traditional diet culture of eating soup and porridge, the intake of which will greatly increase the intake of water in food [32].

One of the findings that attracted our attention in this study was that 16.4% and 43.8% of pregnant women achieved the TWI recommendation set by the CNS and the EFSA, respectively, which is similar to the results of pregnant women in Beijing [14] and young adults in Baoding [32], China. Which TWI recommendation is suitable for Chinese pregnant women? Based on our results, we cannot determine whether pregnant women are in a dehydrated state, only according to whether the amount of TWI meets the recommendation because there is insufficient data on biomarkers of the state of hydration [33]. However, this finding suggests that the relationship between drinking patterns and hydration in pregnant women should be studied further to provide a basis for revising the AI of TWI for Chinese pregnant women. In addition, we found that, similar to a previous study on Chinese young adults [34], the contribution of the water from beverages was higher than that of the water from foods in the pregnant women whose TWI reached the AI set by CNS and EFSA, while the contribution of the water from beverages was almost equal to that of the water from foods in the pregnant women whose TWI did not meet the relevant recommendations. This finding indicated that pregnant women who did not achieve the recommended TWI should increase the amount of water they consume through beverages. Our study found that 80.5% of the water from beverages in Chinese pregnant women was mainly from plain water (highly correlated, *r* = 0.973), which was higher than that of pregnant women in Indonesia [21] (72%), Greece [19] (50%) and Mexico [20] (33%). The difference between different countries may be caused by drinking habits and specific preferences for specific flavors and drinks of individuals. Drinking plain water, such as tap water or bottled water, provides hydration and satiety without the addition of calories [35]. In particular, when substituted for SSDs, it may help to control body weight and improve dietary quality, which is very important for the health of both mothers and infants during pregnancy and can reduce the risk of gestational complications such as gestational diabetes [36]. Therefore, pregnant women whose TWI does not meet the recommendation should first increase the intake of plain water, which is very easy to achieve in China due to their drinking habits.

Many SSDs and FVDs contain large amounts of sugar. Therefore, the contribution of beverages to the TEI was also considered in this study. Our results showed that 9.7% of the daily TEI of Chinese pregnant women was provided by beverages. However, relevant results in different countries are inconsistent. For example, previous studies on female adults in Italy [25], France [26], the Balearic Islands [27] and Spain [28] showed that 5%, 7.8%, 9.5% and 11.6%, respectively, of daily TEI were provided by beverages. In our study, MMDs had the highest contribution to energy from beverages, accounting for approximately 6.9% of the TEI, which was relatively close to the values reported by previous studies on female adults in the Balearic Islands (4.5%) and Spain (6.0%). In contrast to our results, in Italy and France, alcoholic beverages contributed the most to the TEI of female adults, accounting for 2.0% and 2.7%, respectively, followed by MMDs (1% and 2.1%, respectively). However, in our study, no pregnant women were found to consume alcoholic beverages, the reason being that the study population is different. Consuming alcoholic beverages during pregnancy will have adverse effects on maternal and child health [37,38]. As expected, energy from beverages was highly correlated with energy from SSDs (*r* = 0.836) in our study. The contribution of BPDs, SSDs, FVDs and hot beverages to the TEI was relatively low (all less than 1%), which might be related to the particularity of the study population and drinking habits.

Our study has some strengths and limitations. In terms of strengths, we recruited a nationwide sample, which was an average distribution in the second and third trimesters of pregnancy and could be generalized for the whole Chinese pregnant population. Furthermore, we used an online diary with a Tablet/iPad/Phone resource to assess the water and dietary intake of pregnant women for four days (including two weekdays and two weekend days) with the help of a food atlas with three visual reference elements, which not only covered 303 types of food and two types of tableware but also covered a variety of different states and forms of food, involving different processing and cooking. All these factors increase the likelihood of an accurate estimation of the water and dietary intake of the participants. However, our study had several limitations. First, this was a cross-sectional survey, not a longitudinal survey that tracks the impact of maternal water intake on maternal and infant health during the entire pregnancy. Therefore, caution should be applied when extrapolating the results obtained from the comparison of pregnant women in the second and third trimesters. Second, the data collection was carried out from November 2018 to February 2019 (winter in China), which means that seasonal factors were not considered. Third, we did not consider the physical activity levels of pregnant women. However, due to the low physical activity level of pregnant women in the second and third trimesters, the change in water intake caused by physical activity is expected to be limited. Forth, although our survey has been scientifically designed and quality controlled, the dietary survey itself is very complex, and this survey focused on a special group of pregnant women. There may be some social desirability bias or other systematic biases that may influence the data reporting. Finally, in the absence of biomarkers of hydration, it is impossible to draw a conclusion regarding the hydration state of the study sample.

## 5. Conclusions

This is the beginning of a nationwide study on the TWI of pregnant women in China, and the information presented here allows for a better understanding of the water intake in the whole Chinese pregnant population and supports evidence-based health recommendations, such as providing a reference for revising the AI of the TWI in Chinese pregnant women. As per the limitations discussed above, in the future, surveys that track the health status of pregnant women and their infants throughout the entire pregnancy and measurement of hydration biomarkers would support the development of policies to support a healthy lifestyle in pregnant women.

## Figures and Tables

**Table 1:** Median daily water intake (mL/day) and contribution of water intake from different sources (%) among Chinese pregnant women in 2018 categorized by age and gestational trimesters.

Variables	Total (*n* = 653)		Age (Years)				Gestational Trimesters			
			<28 (*n* = 292)		≥28 (*n* = 361)		Second Trimester (*n* = 307)		Third Trimester (*n* = 346)	
	Median (P25–P75)	%	Median (P25–P75)	%	Median (P25–P75)	%	Median (P25–P75)	%	Median (P25–P75)	%
Total water intake	2190 (1719–2710)	-	2147 (1630–2695)	-	2200 (1811–2767)	-	2189 (1691–2693)	-	2192 (1750–2745)	-
Total fluid intake	1165 (798–1652)	-	1115 (763–1623)	-	1218 (835–1665)	-	1140 (795–1575)	-	1196 (800–1690)	-
Water from beverages	1145 (774–1606)	52.9	1086 (742–1599)	52.7	1191 (805–1626)	53.0	1113 (768–1547)	52.9	1177 (781–1663)	52.9
Plain water	1000 (600–1450)	80.5	800 (600–1200)	80.5	1000 (600–1500)	80.5	900 (600–1200)	80.7	1000 (600–1500)	80.3
Hot beverages	0 (0–0)	0	0 (0–0)	0	0 (0–0)	0	0 (0–0)	0	0 (0–0)	0
MMDs	147 (68–234)	14.7	147 (66–223)	14.6	147 (68–252)	14.9	152 (67–225)	14.6	146 (68–237)	14.8
FVDs	0 (0–0)	0.8	0 (0–0)	0.7	0 (0–0)	0.8	0 (0–0)	0.6	0 (0–0)	0.9
SSDs	0 (0–0)	1.4	0 (0–0)	1.5	0 (0–0)	1.3	0 (0–0)	1.4	0 (0–0)	1.3
BPDs	0 (0–50)	2.6	0 (0–50)	2.7	0 (0–46)	2.5	0 (0–50)	2.6	0 (0–46)	2.7
Water from foods	1011 (759–1259)	47.1	969 (700–1281)	47.3	1023 (795–1242)	47.0	1023 (745–1276)	47.1	1004 (768–1236)	-
Staple food	202 (147–302)	23.5	201 (146–305)	23.9	202 (148–297)	23.1	201 (146–294)	23.6	202 (147–308)	23.4
Dishes	313 (210–415)	32.4	280 (190–391) *	31.0 ^#^	325 (228–432)	33.5	311 (200–401)	31.7	314 (215–428)	33.0
Porridge	108 (0–239)	14.7	106 (0–258)	15.5	108 (0–230)	14.0	116 (0–256)	15.2	102 (0–224)	14.3
Soup	125 (53–225)	14.6	128 (50–225)	15.1	125 (61–219)	14.2	125 (51–225)	14.2	126 (55–225)	14.9
Snacks	138 (79–214)	14.9	133 (76–212)	14.5	141 (81–218)	15.2	141 (83–217)	15.4	134 (73–209)	14.5

P25: the 25th percentile; P75: the 75th percentile; %: calculated by average; MMDs: milk and milk derivatives; FVDs: fruit and vegetables drinks; SSDs: sugar sweetened drinks; BPDs: botanical protein drinks; * Statistically significant difference between groups in the median daily water intake (mL/day) of different sources; *p* < 0.05; ^#^ Statistically significant difference between groups in the contribution of water intake from different sources to total water intake (%), *p* < 0.05.

**Table 2:** Median daily water intake (mL/day) and contribution of water intake from different sources (%) among Chinese pregnant women in 2018 categorized by adequate intakes of total water intake set by the Chinese Nutrition society or European Food Safety Authority.

Variables	Comparison of Actual TWI with Water AI from CNS				Comparison of Actual TWI with Water AI from EFSA			
	Group C1 (*n* = 107, 16.4%)		Group C2 (*n* = 546, 83.6%)		Group E1 (*n* = 286, 43.8%)		Group E2 (*n* = 367, 56.2%)	
	Median (P25–P75)	%	Median (P25–P75)	%	Median (P25–P75)	%	Median (P25–P75)	%
Total water intake	3425 (3130–3628)	-	2057 (1648–2425) *	-	2805 (2528–3222)	-	1817 (1473–2060) *	-
Total fluid intake	2100 (1834–2413)	-	1063 (742–1365) *	-	1700(1342–2052)	-	874 (645–1138) *	-
Water from beverages	2071 (1799–2386)	60.7	1044 (711–1320) *	51.4 ^#^	1673 (1309–2036)	57.2	854 (630–1112) *	49.5 ^#^
Plain water	1800 (1500–2000)	86.3	800 (500–1000) *	79.4 ^#^	1500 (1000–1800)	82.5	700 (500–1000) *	78.9 ^#^
Hot beverages	0 (0–0)	0.0	0 (0–0)	0.0	0 (0–0)	0.0	0 (0–0)	0.0
MMDs	181 (108–318)	10.8	138 (56–225) *	15.5 ^#^	191 (106–305)	13.7	117 (49–189) *	15.6 ^#^
FVDs	0 (0–0)	0.6	0 (0–0)	0.8	0 (0–0)	0.7	0 (0–0)	0.8
SSDs	0 (0–0)	0.8	0 (0–0)	1.5 ^#^	0 (0–0)	1.1	0 (0–0)	1.6
BPDs	0 (0–52)	1.6	0 (0–47)	2.8 ^#^	0 (0–47)	2.0	0 (0–39)	3.1 ^#^
Water from foods	1330 (1103–1572)	39.3	952 (730–1172) *	48.6 ^#^	1224 (1006–1449)	42.8	857 (676–1062) *	50.5 ^#^
Staple food	254 (185–356)	21.5	192 (140–287) *	23.9 ^#^	252 (166–365)	23.0	180 (129–258) *	23.9
Dishes	405 (261–524)	30.6	298 (201–397) *	32.7	361 (251–485)	31.0	272 (192–369) *	33.4 ^#^
Porridge	208 (13–420)	16.7	103 (0–223) *	14.3	138 (0–326)	15.0	96 (0–211) *	14.5
Soup	200 (124–290)	17.4	120 (50–200) *	14.0 ^#^	169 (97–271)	15.9	100 (40–188) *	13.6 ^#^
Snacks	164 (125–234)	13.8	130 (73–209) *	15.1	167 (110–247)	15.2	117 (62–173) *	14.7

P25: the 25th percentile; P75: the 75th percentile; %: calculated by average; TWI: total water intake; AI: adequate intake; CNS: Chinese Nutrition Society; EFSA: European Food Safety Authority; MMDs: milk and milk derivatives; FVDs: fruit and vegetables drinks; SSDs: sugar sweetened drinks; BPDs: botanical protein drinks. Group C1: the group whose daily TWI reached the AI value set by CNS; Group C2: the group whose daily TWI did not reach the AI value set by CNS; Group E1: the group whose daily TWI reached the AI value set by EFSA; Group E2: the group whose daily TWI did not reach the AI value set by EFSA; * Statistically significant difference between groups in the median daily water intake (mL/day) of different sources, *p* < 0.05; ^#^ Statistically significant difference between groups in the contribution of water intake from different sources to total water intake (%), *p* < 0.05.

**Table 3:** Median daily energy intake (kcal/day) and contribution of energy intake from different sources (%) among Chinese pregnant women in 2018 categorized by age and gestational trimesters.

Variables	Total (*n* = 653)		Age (Years)				Gestational Trimesters			
			<28 (*n* = 292)		≥28 (*n* = 361)		Second Trimester (*n* = 307)		Third Trimester (*n* = 346)	
	Median (P25–P75)	%	Median (P25–P75)	%	Median (P25–P75)	%	Median (P25–P75)	%	Median (P25–P75)	%
Total energy intake	1589 (1311–1915)	-	1528 (1256–1869) *	-	1639 (1370–1964)	-	1597 (1294–1918)	-	1583 (1324–1908)	-
Energy from beverages	128 (70–211)	9.7	124 (72–200)	9.8	132 (70–220)	9.6	123 (68–208)	9.3	131 (71–211)	10.1
Plain water	0 (0–0)	0	0 (0–0)	0	0 (0–0)	0	0 (0–0)	0	0 (0–0)	0
Hot beverages	0 (0–0)	0.3	0 (0–0)	0	0 (0–0)	0.5	0 (0–0)	0.2	0 (0–0)	0.4
MMDs	96 (45–156)	71.3	92 (41–143)	70.5	99 (45–164)	72.0	94 (44–151)	72.4	97 (45–162)	70.4
FVDs	0 (0–0)	5.2	0 (0–0)	4.5	0 (0–0)	5.7	0 (0–0)	4.0 ^#^	0 (0–0)	6.2
SSDs	0 (0–0)	10.1	0 (0–0)	10.5	0 (0–0)	9.9	0 (0–0)	9.0	0 (0–0)	11.2
BPDs	0 (0–16)	10.2	0 (0–16)	10.4	0 (0–14)	10.0	0 (0–16)	10.5	0 (0–14)	9.8
Energy from foods	1420 (1186–1703)	90.3	1343(1123–1649) *	90.2	1477 (1244–1729)	90.4	1393 (1177–1708)	90.7	1429 (1188–1691)	89.9
Staple food	447 (309–588)	31.5	423 (288–567)	31.0	463 (340–610)	31.9	447 (308–590)	31.4	446 (309–585)	31.6
Dishes	713 (578–861)	50.3	690 (561–825) *	50.8	728 (588–875)	49.9	713 (575–844)	49.9	715 (578–869)	50.6
Porridge	14 (0–48)	2.7	13 (0–47)	2.8	14 (0–49)	2.6	19 (0–52)	2.7	12 (0–46)	2.7
Soup	0 (0–0)	0	0 (0–0)	0	0 (0–0)	0	0 (0–0)	0	0 (0–0)	0
Snacks	205 (114–329)	15.6	197 (107–324)	15.5	211 (133–333)	15.7	217 (123–333)	16.1	199 (109–326)	15.2

P25: the 25th percentile; P75: the 75th percentile; %: calculated by average; MMDs: milk and milk derivatives; FVDs: fruit and vegetables drinks; SSDs: sugar sweetened drinks; BPDs: botanical protein drinks; * Statistically significant difference between different groups in the median daily energy intake (kcal/day) of different sources, *p* < 0.05; ^#^ Statistically significant difference between different groups in the contribution of energy intake from different sources to total energy intake (%), *p* < 0.05.

**Table 4:** Partial correlation between the water and energy intake of different sources and total water intake, total fluid intake, water from beverages and foods, total energy intake, energy from beverages and foods (adjusted for age and gestational period).

**Variables**	**Total Water Intake**	**Total Fluid Intake**	**Water from Beverages**	**Water from Foods**	**Total Energy Intake**	**Energy from Beverages**	**Energy from Foods**
Total water intake	1.000	0.874 ***	0.871 ***	0.609 ***	0.433 ***	0.225 ***	0.402 ***
Total fluid intake	0.874 ***	1.000	1.000 ***	0.146 ***	0.170 ***	0.224 ***	0.108 **
Water from beverages	0.871 ***	1.000 ***	1.000	0.141 ***	0.160 ***	0.209 ***	0.102 ***
Plain water	0.829 ***	0.966 ***	0.973 ***	0.099 *	0.063	0.057	0.049
Hot beverages	0.005	−0.014	−0.015	0.034	0.037	0.046	0.025
MMDs	0.361 ***	0.362 ***	0.340 ***	0.179 ***	0.290 ***	0.434 ***	0.165 ***
FVDs	0.116 **	0.081 *	0.067	0.125 **	0.294 ***	0.383 ***	0.188 ***
SSDs	0.030	0.020	0.016	0.034	0.327 ***	0.725 ***	0.098 *
BPDs	0.083 *	0.093 *	0.094 *	0.017	0.062	−0.026	0.079 *
Water from foods	0.609 ***	0.146 ***	0.141 ***	1.000	0.616 ***	0.117 **	0.647 ***
Staple food	0.289 ***	0.037	0.036	0.526 ***	0.388 ***	−0.022	0.442 ***
Dishes	0.391 ***	0.085 *	0.078 *	0.663 ***	0.560 ***	0.115 **	0.585 ***
Porridge	0.213 ***	−0.016	−0.013	0.450 ***	0.064	−0.044	0.088 *
Soup	0.384 ***	0.190 ***	0.185 ***	0.475 ***	0.197 ***	0.148 ***	0.166 ***
Snacks	0.325 ***	0.128 **	0.122 **	0.457 ***	0.457 ***	0.161 ***	0.453 ***
Total energy intake	0.433 ***	0.170 ***	0.160 ***	0.616 ***	1.000	0.472 ***	0.946 ***
Energy from beverages	0.225 ***	0.224 ***	0.209 ***	0.117 **	0.472 ***	1.000	0.160 ***
Plain water	-	-	-	-	-	-	-
Hot beverages	0.030	0.037	0.032	0.009	0.039	0.067	0.019
MMDs	0.364 ***	0.356 ***	0.334 ***	0.195 ***	0.305 ***	0.444 ***	0.178 ***
FVDs	0.102 **	0.073	0.059	0.111 **	0.257 ***	0.365 ***	0.153 ***
SSDs	0.014	0.023	0.021	−0.005	0.311 ***	0.836 ***	0.040
BPDs	0.083 *	0.093 *	0.094 *	0.017	0.062	−0.026	0.079 *
Energy from foods	0.402 ***	0.108 **	0.102 **	0.647 ***	0.946 ***	0.160 ***	1.000
Staple food	0.174 ***	0.000	0.000	0.350 ***	0.559 ***	−0.032	0.638 ***
Dishes	0.342 ***	0.101 *	0.094 *	0.538 ***	0.722 ***	0.176 ***	0.744 ***
Porridge	0.145 ***	0.027	0.028 ***	0.248 ***	0.158 ***	0.018	0.170 ***
Soup	-	-	-	-	-	-	-
Snacks	0.268 ***	0.121 **	0.113 **	0.357 ***	0.628 ***	0.191 ***	0.633 ***

MMDs: milk and milk derivatives; FVDs: fruit and vegetables drinks; SSDs: sugar sweetened drinks; BPDs: botanical protein drinks. * *p* < 0.05; ** *p* < 0.01; *** *p* < 0.001.

## Data Availability

The data presented in this study are available on request from the corresponding author.

## Supplementary Materials

The following are available online at https://www.mdpi.com/article/10.3390/nu13072219/s1, Table S1: Classification of the beverage types, Table S2: Classification of the food types.
