# Supplementary material for: Water Intake in Pregnant Women in China, 2018: The Report of a Survey
Source: Nutrients. 2021 Jun 28;13(7):2219. doi: 10.3390/nu13072219 (PMC8308335; doi:10.3390/nu13072219)
Supplement: Supplementary file 1 [file nutrients-13-02219-s001.zip › nutrients-1272204-SI.pdf]

nutrients MDPI

Table S1. Classification of the beverage types

Classification	Detailed beverage types
**Plain water**	Tap water, Bottled water (mineral water, purified water).
**Hot beverages**	Coffee (coffee from coffee maker, powder coffee, instant coffee, etc), Tea (black tea, green tea, flower tea, etc).
**Milk and milk derivatives**	Liquid milk (low fat milk, full fat milk), Powdered milk (whole or skimmed powdered milk, powdered milk for pregnant women), Yoghurt, etc.
**Fruit and vegetables drinks**	100% Fruit juice (bottled or freshly squeezed watermelon juice, pear juice, orange juice, etc), 100% Fruit and vegetable juice (bottled or freshly squeezed cucumber and pineapple mixed juice, orange and carrot mixed juice, apple and celery mixed juice, etc).
**Sugar sweetened drinks**	Carbonated beverages (Cola, Sprite, Fanta, etc), Tea flavoured beverage (bottled/boxed ice black tea, ice green tea, jasmine tea, etc), Fruit flavored beverage (peach flavored, lemon flavored, grapefruit flavored, etc), Milk flavored beverage (chocolate milk, banana milk, coconut milk, etc), Functional beverages (probiotics, dietary fiber, vitamins, etc), Milky tea (pearl milky tea, coconut jelly milk tea, fruit milk tea, etc), etc.
**Botanical protein drinks**	Soybean milk, almond juice, walnut juice, etc.

nutrients MDPI

Table S2. Classification of the food types 1

Classification	Detailed food types
**Staple food**	
Cereals and cereals products	Rice, Steamed buns, Bread, Steamed twisted rolls, Fried dough sticks, Noodles, etc.
Compound processed products	Dumpling, Wonton, Hamburger, Sandwich, etc.
Others	Sweet potato, Starch vermicelli, etc.
**Dishes**	
Vegetables	Spinach, Cabbage, Bok choy, Leaf lettuce, Chinese leek, Garlic bolt, Cauliflower, Broccoli flower, Chinese cabbage, Carrot, Potato, Chinese yam, Taro, Bamboo shoot, Onion, Garlic, Lotus root, Cucumber, Summer squash, Towel gourd, Pumpkin, White gourd, Bitter gourd, Eggplant, Cowpea, Snap bean, Snow pea, Green soybean, Pea, Soybean sprout, Green gram sprouts, Mushroom, Laver, Kelp seaweed, etc.
Beans and legume products	Tofu, Shredded tofu, Dried bean curd, Tofu skin, etc.
Aquatic products	Grass carp, Catfish, Loach, Carp, Yellow croaker, Perch, Sea shrimp, River shrimp, Lobster, Sea crab, River crab, Oysters, Clams, Scallops, Cuttlefish, Octopus, etc.
Livestock meat	Pork, Pork (heart / ear / liver / kidney / blood / intestine), Trotters, Bacon, Lunch meat, Beef, Beef tongue, Tripe, Mutton, Lamb liver, Lamb belly, etc.
Poultry	Chicken, Chicken (wings / legs / claw / liver / heart / gizzards / blood), Duck, Duck (wings / legs / claw / tongue / intestine / liver / gizzard / blood), Goose, Goose (wings / legs / liver / gizzard / blood), Quail, Pigeon, etc.
Eggs	Hen's egg, Duck egg, Goose egg , Quail egg, etc.
**Porridge**	Rice porridge, Millet porridge, Corn porridge, Black rice and mixed bean porridge, etc.
**Soup**	Tomato egg drop soup, Oyster soup, etc.
**Snacks**	
Fruits	Apple, Pear, Hawthorn, Peach, Apricot, Plum, Papaya, Cherry, Grape, Persimmon, Tangerines, Grapefruit, Watermelon, Honeydew melon, Strawberry, Kiwifruit, Mulberry, Banana, Mango, Litchi, Pitaya, Durian, etc.
Nuts	Walnut, Chestnut, Pine nut, Peanut, Sunflower seed, Pumpkin seed, watermelon seed, etc.
Others	Cake, Cookies, Chocolate, Ice cream, etc.
